# Mixed Effects of Habitat Degradation and Resources on Hantaviruses in Sympatric Wild Rodent Reservoirs within a Neotropical Forest

**DOI:** 10.3390/v13010085

**Published:** 2021-01-09

**Authors:** Jeremy V. Camp, Briana Spruill-Harrell, Robert D. Owen, Carles Solà-Riera, Evan P. Williams, Gillian Eastwood, Aubrey M. Sawyer, Colleen B. Jonsson

**Affiliations:** 1 Institute of Virology, University of Veterinary Medicine Vienna, 1210 Vienna, Austria; Jeremy.Camp@meduniwien.ac.at; 2 Department of Microbiology, Immunology and Biochemistry, University of Tennessee Health Science Center, Memphis, TN 38163, USA; bspruill@uthsc.edu (B.S.-H.); ewilli99@uthsc.edu (E.P.W.); 3 Centro para el Desarrollo de la Investigación Científica, Asunción C.P. 1371, Paraguay; rowen@pla.net.py; 4 Department of Biological Sciences, Texas Tech University, Lubbock, TX 79409, USA; 5 Center for Infectious Medicine, Department of Medicine Huddinge, Karolinska Institutet, Karolinska University Hospital, 141 86 Stockholm, Sweden; carles.sola.riera@ki.se; 6 Department of Microbiology, University of Tennessee-Knoxville, Knoxville, TN 37996, USA; gill2g@hotmail.com (G.E.); asawye11@vols.utk.edu (A.M.S.)

**Keywords:** dilution effect, hantaviruses, interior Atlantic Forest, resource augmentation, species diversity

## Abstract

Understanding the ecology of rodent-borne hantaviruses is critical to assessing the risk of spillover to humans. Longitudinal surveys have suggested that hantaviral prevalence in a given host population is tightly linked to rodent ecology and correlates with changes in the species composition of a rodent community over time and/or habitat composition. We tested two hypotheses to identify whether resource addition and/or habitat composition may affect hantavirus prevalence among two sympatric reservoir hosts in a neotropical forest: (i) increased food resources will alter the rodent community and thus hantaviral prevalence; and (ii) host abundance and viral seroprevalence will be associated with habitat composition. We established a baseline of rodent–virus prevalence in three grid pairs of distinct habitat compositions and subjected one grid of each pair to resource augmentation. Increased rodent species diversity was observed on grids where food was added versus untreated control grids during the first post-treatment sampling session. Resource augmentation changed species community composition, yet it did not affect the prevalence of hantavirus in the host population over time, nor was there evidence of a dilution effect. Secondly, we show that the prevalence of the virus in the respective reservoir hosts was associated with habitat composition at two spatial levels, independent of resource addition, supporting previous findings that habitat composition is a primary driver of the prevalence of hantaviruses in the neotropics.

## 1. Introduction

Rodent-borne hantaviruses (Family *Hantaviridae*, Genus *Orthohantavirus*) are maintained by transmission within reservoir rodent species across complex ecosystems in diverse landscapes and habitats across the globe [1]. We know that ecological factors influence the spillover of hantaviruses to new hosts, such as other rodent species or humans, where infection may cause severe diseases such as hantavirus pulmonary syndrome (HPS) or hemorrhagic fever with renal syndrome (HFRS). Few field studies have experimentally addressed hypotheses to test mechanisms proposed to promote the maintenance, spillover or emergence of hantaviruses harbored by reservoir species within a rodent community within its native ecosystem [2,3]. Longitudinal, descriptive studies of rodent populations have suggested that several ecological factors correlate with the increased prevalence of hantaviruses, such as tree mast seeding [4,5,6], lower rodent species diversity [2,3], decreased predator abundance [7,8,9], climatic events [10,11,12,13,14,15], habitat fragmentation [16], or land cover classes [17]. Hence, the following question arises: do these ecological factors similarly influence hantavirus prevalence among their respective rodent communities across diverse ecosystems (e.g., Desert Southwest versus a European forest)? In other words, are there common drivers of hantavirus prevalence within reservoir species, independent of ecosystem, and are there similar mechanisms which promote emergence and spillover into humans?

Human epidemics of nephropathia epidemica, a mild form of HFRS, follow seasonal and multiannual fluctuations preceded by high seroprevalence of *Puumala orthohantavirus* (PUUV) in bank vole (*Myodes glareolus*) populations in Europe [18]. The bank vole, the natural reservoir of PUUV, predominates in oak and beech forests from Western Russia into Central Europe, where hot summers and mild winters promote tree growth [19]. Peaks of oak (*Quercus robur* and *Q. petraea*) and beech (*Fagus sylvatica*) mast density have been shown to promote rodent survival and reproduction a year later and strongly correlate with incidence of HFRS [20,21,22,23]. Similarly, it was postulated that mast seeding of the piñon pine in the Desert Southwest, USA, following the 1991–1992 El Niño event drove the expansion of deer mouse (*Peromyscus maniculatus*) populations and increased the prevalence of *Sin Nombre orthohantavirus* (SNV) [15,24]. Similar to the oak–beech forests of Europe, there is low tree species diversity in the semi-arid woodlands in the American Southwest, where piñon pine, juniper, and oak predominate [25]. In contrast, the boreal forests of Northern Europe have smaller seasonal variations in seed production, and rodent population levels depend also on predator–prey interactions [7,8,9,18]. This biogeographic region has been extensively studied, and longitudinal observations of hantavirus abundance are statistically associated with rodent abundance given certain population characteristics during relatively short time frames (i.e., a young, “naïve” population is associated with increased hantavirus prevalence, but not necessarily total rodent population) [26].

In studies conducted in the American tropics (the neotropics), spanning from Mexico to southern South America, the dynamics of hantaviral seroprevalence have not been as strongly associated with climatic factors or mast seeding, but instead with landscape composition, landscape topology, landscape fragmentation, and biogeographic history [16,17,27,28,29,30,31]. This is reasonable given that the neotropical rainforests differ from the American Southwest or forests of Europe in that they have two to three seasons annually based on precipitation patterns, with relatively small differences in temperature throughout the year, and are the most species-rich regions on Earth, resulting in a highly complex biotic community. In Brazil, anthropogenic landscape change promotes the opportunistic and aggressive behavior of the rodent *Necromys lasiurus* and this may be an important factor in driving spillover of the *Araraquara orthohantavirus* to humans [28,32,33]. In the neotropical forests of Paraguay, the highest levels of hantaviral seroprevalence occur in moderately disturbed forest areas and are lower in highly disturbed sites (i.e., pastures) and in pristine forested areas [17]. In the Atlantic Forest within Paraguay and Brazil, the structure of the rodent community changes spatially, in response to land cover composition [32,34]. Given that rodents are tied ecologically to specific habitats, in which movement is limited to a radius that is generally much less than 1 km, degradation or alterations of rodent habitat may result in increased movement and contact, thereby leading to increased transmission and higher viral prevalence. This suggests that rodent habitats undergoing modification or with certain characteristics of ecological disturbance are important sites for the increased contact, spillover, and spread of the virus in rodent reservoirs.

The drivers that increase the prevalence or spread of hantaviruses in their reservoirs in nature may be significant alone or act in concert. In the neotropical forests of South America, it is clear that the outcome depends primarily on the ecology of rodent–virus interactions within their immediate local environment. We have identified and characterized an ecosystem in a neotropical forest in Paraguay where *Akodon montensis* and *Oligoryzomys nigripes*, two sigmodontine rodents, are the primary reservoirs of Jabora and Juquitiba viruses, respectively [34,35,36,37,38]. This system allows us to investigate ecological factors that influence hantavirus prevalence and test whether those factors are species-specific. Experimentally altering habitat effects and/or species diversity directly is problematic in field experiments; we therefore altered the availability of resources to simulate mast seeding in temperate forests, a factor that is generally accepted to increase hantavirus prevalence in rodents in the short term [5,6,19,21,25]. We added resources to specific rodent sampling grids to test the hypothesis that an increase in food resources would alter the rodent community, and then to analyze the effects on the two principal hantavirus reservoir species and the rodent community.

As we have intensively characterized landscape effects on rodent dynamics within this habitat [17,34,35,36,37,38,39,40,41,42], we could control for landscape- and habitat-specific effects which may otherwise confound studies in the neotropics [14,16,37,39,43,44]. This allowed us to control for (and test) the previous observations that habitat degradation may be the primary driver of hantavirus prevalence in neotropical habitats [36,37,40,41,42]. More specifically, we have previously shown that these two rodent reservoir species have fundamentally different microhabitat requirements, defined by vegetation characteristics at a given trap station, yet are sympatric on a marginally larger spatial scale [36,37]. This enabled us to test whether these changes in resource augmentation affected fine-scale habitat associations of rodent reservoir abundance and hantavirus prevalence as well as our previously defined larger scale associations of habitat degradation [17,40]. To our knowledge, no study of hantavirus prevalence has determined the presence of hantavirus-infected rodents on a fine-scale habitat level.

The ultimate goal of basic research into enzootic virus transmission and maintenance is to infer the likelihood of epizootic transmission. Therefore, we consider whether spillover infections to other rodent species (non-reservoir species) may serve as an indication of the likelihood of spillover to humans, but we do not test that directly here. We present a cross-sectional, short-term study of the prevalence of two hantaviruses within a focus of transmission and analyze hantavirus prevalence with respect to experimental manipulation (resource augmentation), experimental control (habitat degradation), and species characteristics (age, sex, etc.). We provide an analysis of rodent occupation and hantaviral presence over several sites with three landscape conditions with and without food augmentation.

## 2. Materials and Methods

### 2.1. Rodent Collection

Grid selection and design, and environmental variables, are described in Supplemental Text. Sherman traps (7.6 × 8.9 × 22.9 cm, Sherman Trap Company™, Tallahassee, FL, USA) were set 10 m apart in a 12 by 12 grid on each site with one above ground and one on the ground. Each of the six grids was run for five nights during each of the three sampling sessions, for a total of 38,880 trap nights. For each capture, the date, grid, station number (row and column), and trap height (on ground or above ground) were recorded. Rodents were identified following [45] and authors therein. The animal’s weight, sex, reproductive status, and age category (juvenile, sub-adult, adult) were also recorded. In July and November of 2015, samples of saliva, blood, urine, feces, and a small (1–2 mm) tail snip were collected, and rodents were tagged with passive integrated transponders and released. In the February sampling, rodents were taken to the field laboratory, where liver, lung, heart, kidney, muscle, spleen, colon, blood, urine, saliva, and embryos (when encountered) were harvested and stored immediately in liquid nitrogen, prior to transport to the University of Tennessee, where the samples were stored at −80 °C until processing. Any clinical signs, such as enlarged spleen, liver parasites, or tail lesions or scars from *Leishmania* spp. Infections, were noted during necropsy.

### 2.2. Resource Augmentation

**Figure 1:**
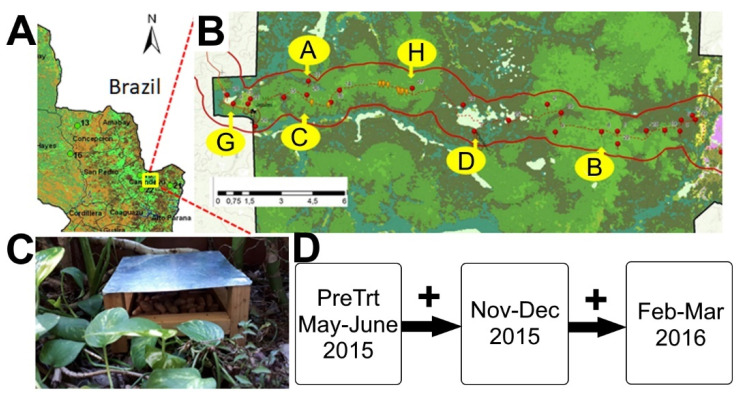
Map of the Mbaracayú Forest Biosphere Reserve (MFBR) and sampling efforts. (**A**) Partial map of Paraguay showing the location of the MFBR in Northeastern Paraguay on the border with Brazil. (**B**) Map showing the locations of the six grids, A, B, C, D, G, and H. Grids were approximately 120 by 120 m in size with 144 unique stations. Sherman traps were placed at each station on the ground and above the ground for a total of 288 for the first session and two on the ground and one above for subsequent post-treatment sessions (432 per grid). Grids B and H were the least disturbed sections of the MFBR, moderately disturbed habitats were found in grids A and D, and grids C and G were the most disturbed areas. (**C**) Example of feeding station used on each of three grids: H, D, and C. Sixteen feeding stations were placed in each grid and refilled weekly during two time periods. (**D**) Timeline of small mammal capture activities during study, where plus symbols (“+”) indicate periods when resources were added to the selected grids (grids H, D, and C).

The study was carried out within the largest protected neotropical forest in Paraguay, the Mbaracayú Forest Biosphere Reserve (MFBR), a 64,405-ha UNESCO Biosphere Reserve. The MFBR comprises the Interior Atlantic Forest, which formerly covered large portions of Southern Brazil, Northeastern Argentina, and Eastern Paraguay. Previously, we surveyed 21 sites across the MFBR (Figure 1A) for rodents and measured the presence of antibodies to hantaviral antigens as well as the presence and sequencing of hantaviral RNA [37]. Based on this information, we extensively assessed vegetation and habitat disturbance to select three pairs of 12 × 12 sampling grids (Supplemental Text, Figure S1). Briefly, we determined habitat degradation based on a principal component analysis of six variables, and pairs of grids were assigned to a habitat degradation category, which were closely grouped in the first two components, based primarily on the presence of invasive trees and overall measures of habitat destruction (Supplemental Text, Figure S1). Three pairs of grids were designated: B and H—least degraded; A and D—moderately degraded; C and G—most degraded (Figure 1B). One grid from each pair—grids H, D and C—was selected as described in the Supplemental Text for resource augmentation.

Sixteen feeding stations per grid were established and provided with a commercial pet food mixture (consisting of whole kernel corn, peanuts in the shell, sunflower seeds, rice, and a compressed pellet of undisclosed ingredients) on a weekly basis as supplemental food. The choice of food was selected following a preliminary field comparison of the consumption of several alternative foods by the target species. Food was heat-treated to prevent germination (see Supplemental Text). Two feeders were placed at each station (one on the ground, and one placed 2–3 m above ground, in such a way that it could be accessed from branches or vines). Feeding stations were constructed such that only small mammals could access the food (Figure 1C). Fifty grams of the selected food was placed in each of the above-ground feeders, and 150 g in the ground-level feeders, on a weekly basis, from 1 August until the beginning of the first post-resource sampling session in late October (ca. 15 weeks), when feeding was discontinued on each grid during the mark-recapture sampling on that grid. Feeding was resumed after the sampling was concluded and continued until the final capture-only sampling session in February 2016 (ca. 11 weeks).

Following an initial pre-treatment baseline survey (June–July 2014 = PreTrt) using the mark–release–recapture (MRR) method, rodents were sampled post-treatment with MRR over five capture nights during November–December 2015 (ND2015), and again after resource augmentation ended in the final capture-only session in February–March 2016 (FM2016) (Figure 1D).

### 2.3. Indirect Immunofluorescence Screen for Antibodies to Hantavirus

An indirect immunofluorescence assay (IFA) was used to screen blood samples for the presence of antibodies cross-reactive with ANDV grown in Vero E6 cells on microscope spot slides as described previously [42]. We have previously demonstrated the sympatry of Jabora virus (JABV) and Juquitiba virus (JUQV) in this study site in their reservoir hosts, *Akodon montensis* and *Oligoryzomys nigripes*, respectively [35,37]. As hantaviruses cause a chronic infection of rodents, we use seroprevalence as a measure of hantaviral activity, as is common in these types of field studies [1,15]. The viruses in this study are genetically similar to ANDV, with 86/96 (N protein) and 93/96 (Gn/Gc) similarity at the amino acid level for JABV/JUQV, respectively, and we have confirmed that this IFA assay can reliably identify seropositive rodents infected with these viruses [35,37,42].

### 2.4. Statistical Methods

All statistical analyses were performed in R version 3.6.2 [46] using the packages cited below. The experimental design was a split-plot design, where resource augmentation was a fixed effect blocking by grids (random effect), and sampling session was a within-grid repeated measures factor. Six habitat variables (i.e., vegetation) were measured at each trapping station to evaluate which grids could be paired as a control and treated grid (Figure S1). Means of these station measures were used to classify each grid according to degradation level, evaluated using both sequential agglomerative hierarchical non-overlapping clustering and principal component analysis with minimum spanning tree (data not shown).

Species alpha diversity was measured by calculating Shannon’s diversity index for each grid session based on unique captures. Linear mixed effect models were used to test for the main effect of resource augmentation on measured variables (e.g., Shannon’s index) according to a partly nested design (package lme4) [47]. Prior to testing, the data were checked for violations of the assumptions of those models (normality, homoscedasticity, and sphericity) using packages “nlme” and “effects” [48,49]. Post hoc tests were performed using variance partitioning and tests of multiple comparisons by Tukey’s or Dunnett’s methods as appropriate using the package “multcomp” [50].

The probability of rodents with antibody to hantavirus was evaluated for the effects of experimental (resource augmentation) or measured variables. First, using univariate logistic regression, we classified the rate of seropositivity in individual rodent species according to sex, age class (juvenile, subadult, adult), weight, presence of a tail scar, or reproductive activity (male with scrotal testes or female pregnant, nursing, or with vagina open). We considered the same rodent captured in different sessions to represent independent data points and ignored recaptures within a given sampling session. Similarly, logistic regression was also used to determine the relationship between experimental variables (resource augmentation, habitat degradation, and habitat clusters). The likelihood ratio was used to compare model fitness to the null model. Simple linear regression was used to test the relationship between proportion of hantavirus-seropositive rodents and Shannon’s index in each grid and session.

Spatially explicit mark–recapture data were analyzed with the package “secr” [51]. Species distribution models were derived from all captures, including recaptures, based on simple generalized linear models with a logit link. Species distribution and predicted probabilities were visualized with the “raster” package [52]. The seven habitat variables mentioned above were used to predict rodent species distribution (Table S3, Figure S6), and five were used to define microhabitat clusters (package “vegan”) (Figure S5) [53]. The habitat variables were highly correlated and therefore a principal component analysis was performed prior to generating the species distribution models (Figure S6). Pre-treatment presence/absence data were used as the training dataset for each of the three most abundant species. The resulting predicted probabilities had bimodal distributions, and grid-specific thresholds were set based on the local minimum between the two modes for training data to create a binary predicted presence/absence at each trap station for each species. These data were then used to test post-treatment observed presence/absences for each species. Model performance and accuracy were assessed using statistics derived from the confusion matrix: specificity and sensitivity were used to calculate a true skill statistic (“TSS” = Specificity + Sensitivity−1) and overall accuracy (the sum of true positives and true negatives divided by the total) [54] (Tables S5 and S6). The mean predicted probability of seronegative individuals was compared to the mean probability of seropositive individuals using Student’s *t*-test with Welch’s correction.

## 3. Results

### 3.1. Cross-Sectional Sampling of the Mbaracayú Forest Biosphere Reserve (MFBR)

A total of 2076 rodent capture events (Table S1), which represented 1165 unique individual rodents (Table 1) and 12 different species, were sampled. These included native Cricetidae (subfamily Sigmodontinae) rodents (*Akodon*, *Calomys*, *Euryoryzomys*, *Holochilus*, *Hylaeamys*, *Nectomys*, *Oligoryzomys*, and *Sooretamys*) and a non-native Muridae rodent (*Rattus*) (Table 1). Of these unique individual rodents, 911 recapture events (between 1 and 10 recaptures per individual) were recorded following the initial encounter. A total of 102 individuals were recaptured in a sampling session after the session in which they were initially encountered. These between-session recaptures were counted as unique individuals per session, which resulted in 1267 individuals being used in subsequent analyses except where noted (e.g., sex could not be determined from 11 individuals, as noted in Table 1). *Akodon montensis* was the most frequently captured rodent (63%) across all grids, followed by *Hylaeamys megacephalus* (19%) and *Oligoryzomys nigripes* (11%) (Table 1).

### 3.2. Population Size, Density, and Experimental Variables

**Figure 2:**
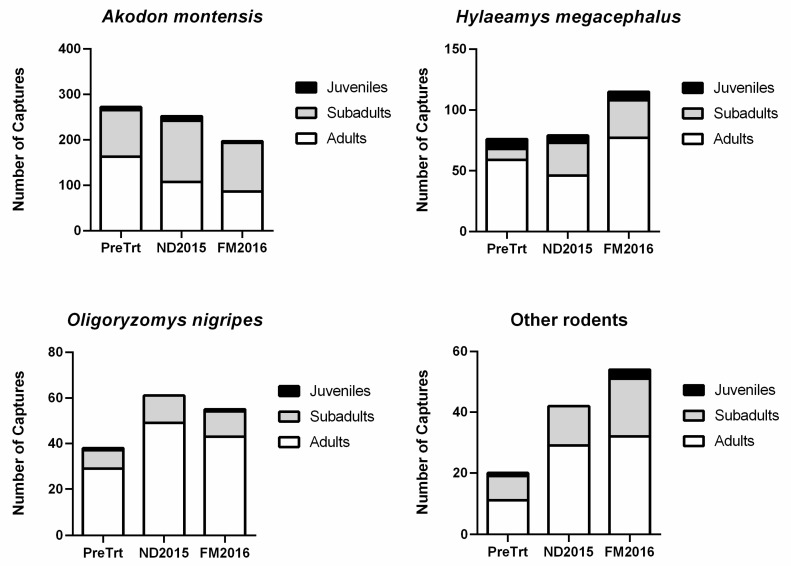
The number of unique captures by age class per session for the three most abundant species and other rodents. The first mark–release–recapture (MRR) session was pre-treatment (PreTrt) sampling performed June–July 2015, which was followed by one post-treatment sampling by MRR conducted November–December 2015 (ND2015) and a second post-treatment sampling by capture conducted February–March 2016 (FM2016). Juveniles are shaded in black, sub-adults are shaded in light gray, and adults are shown in white.

Population sizes were examined for the most abundant species, *A. montensis*, *H. megacephalus*, and *O. nigripes*. All species displayed similar variations in estimated population sizes, with more unique captures during the first post-treatment session (ND2015) and fewer during the second post-treatment session (FM2016) (Figure 2). A higher capture ratio of juveniles and/or sub-adults was observed in the first post-treatment session for the three most abundant species (Figure 2); however, no juveniles were captured in the first post-treatment session for *O. nigripes*. We found no statistical difference in the capture ratio of male and female unique individual rodents (Table 1).

As the study design was a mark–release–recapture study, we used spatially explicit capture–recapture methods to estimate the density and rodent movement on each grid. Ignoring sample session and treatment cofactors, there was little variation among grids for the density and movement of populations of *A. montensis* and *H. megacephalus* (coefficient of variation <30%); however, higher variation was found for populations of *O. nigripes*. To determine if this variation was due to resource augmentation, we modeled the density and movement of these three species using this treatment as a cofactor. Grids with resource augmentation had significantly higher densities of *O. nigripes* (*p* = 0.037) and *H. megacephalus* (*p* = 0.004) compared to grids without resource augmentation. In contrast, the density of *A. montensis* was significantly lower on grids with resource augmentation compared to grids without resource augmentation (*p* = 0.02) (Table S2).

### 3.3. Resource Augmentation and Community Composition

We used hierarchical clustering to visualize community structure using Ward’s distance to group each grid session by Jaccard’s dissimilarity index (Figure S2). With some exceptions, grids which received resource augmentation were generally similar to each other in terms of species composition and relative abundance, and treatment appeared to have increased the abundance of some otherwise less abundant species. Therefore, we were interested in whether resource augmentation affected species diversity.

**Figure 3:**
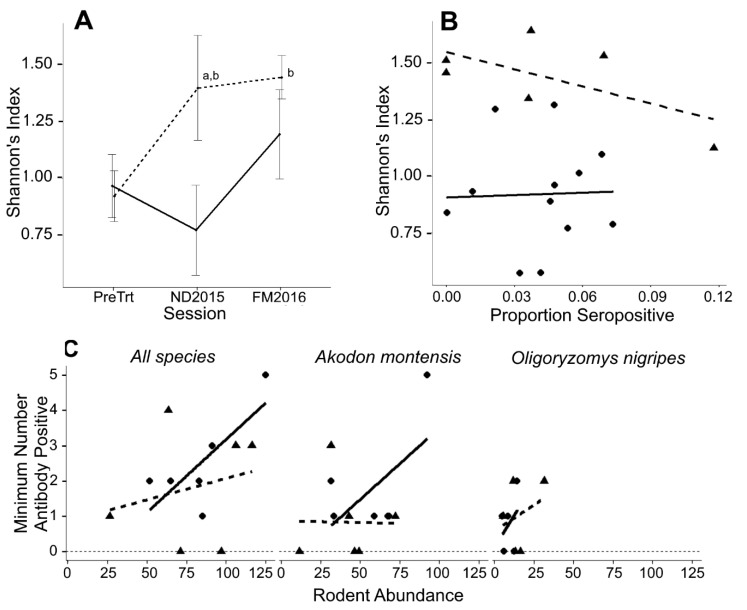
**The effects of resource augmentation on species diversity, rodent abundance, and seroprevalence.** (**A**) Following the pre-treatment sampling period (PreTrt), resources were added to three of the six grids and rodents were sampled twice: post-treatment sampling by MRR, ND2015, and post-treatment sampling by capture, FM2016. Shannon’s index was calculated based on all unique captures per grid, per sampling period. (a) Diversity on treated grids (triangles and dashed lines) was significantly higher than control grids (circles and solid lines) at the first post-treatment sampling session, ND2015; and (b) diversity on treated grids was significantly higher during both post-treatment sampling sessions compared to pre-treatment sampling session. (**B**) There was no relationship between species diversity and seroprevalence on untreated (circles, solid least squares line) or on treated grids (triangles, dashed least squares line), as a test of the dilution effect. (**C**) There was no statistically significant association between the estimated rodent abundance based on MRR and seroprevalence for all species, or for the two principal reservoir species, *Akodon montensis* and *Oligoryzomys nigripes* (symbols and lines follow panel B).

The effect of resource augmentation on rodent species diversity (as measured by Shannon’s index, H) was analyzed using linear mixed effect models and Type III repeated-measures ANOVA. Testing model assumptions revealed no violations of normality, homoscedasticity, or sphericity. The main effect of resource augmentation (F_1,4_ = 8.69, *p* = 0.042), the within-block effect of capture session (F_2,8_ = 9.30, *p* = 0.008), and the interaction between resource augmentation and capture session (F_2,8_ = 7.32, *p* = 0.016) significantly explained the variance in Shannon’s index. The within-block variance (i.e., within-grid) accounted for 32.9% of the total residual variability. The value of the Shannon’s index on resource-augmented grids increased 0.67 units (95% C.I. = 0.27~1.08) above PreTrt non-augmented grids during the first post-treatment sampling session (based on linear mixed model coefficient, *p* = 0.005) (Figure 3A). Since the interaction term was significant, the analysis was split to test if variance in Shannon’s index was explained by resource augmentation at a given session. Shannon’s index on augmented grids (mean = 1.40, 95% C.I. = 1.12~1.68) was significantly higher than grids without resource augmentation during the first post-treatment capture session (mean = 0.79, 95% C.I. = 0.54~1.05; Tukey’s post hoc multiple comparisons testing *p* =0.024; Figure 3A). Similarly, comparing each treatment group separately, the Shannon’s index at both post-treatment sessions on resource-augmented grids was higher than PreTrt session (Dunnett’s post hoc test comparing ND2015 and FM2016 to PreTrt, *p* = 0.018 and 0.012, respectively), but there was no significant difference in Shannon’s index between the three sessions on grids without augmentation (Figure 3A). A similar analysis was performed to test if habitat degradation was statistically associated with species diversity; however, we found no association between habitat degradation and species diversity (F_2,15_ = 0.801, *p* = 0.467). A multivariable model was used to determine whether the association between resource augmentation and the Shannon’s index varied according to habitat degradation; however, this model was not a better fit with the data and habitat degradation added no explanatory power to the significant association between Shannon’s index and resource augmentation (not shown). Thus, resource augmentation was a predictor of species diversity independently of habitat degradation.

### 3.4. Statistical Classification of Antibody-Positive Rodents

Rodent blood was screened for antibodies using an indirect immunofluorescence assay (IFA). Of the 1267 unique session captures, blood was analyzed from 944 samples and 41 cross-reacted with ANDV antigen (i.e., seropositive) in the IFA test: 14 in the PreTrt session, 12 in the first period post-treatment ND2015, and 15 in the second period post-treatment FM2016 (Table 2). The analyzed samples were taken from 887 unique individuals, 57 of which were sampled and tested in more than one sampling session, and six rodents seroconverted during the study (three *A. montensis* and three *O. nigripes*). Of the three species with the highest abundance, *O. nigripes* had the highest prevalence of antibodies, with 14 of 123 (11.38%) individuals (Table 2). *Akodon montensis* had the second highest prevalence of antibodies (*n* = 24 of 527 tested, 4.39%, Table 2). Cross-reactive antibodies were detected by IFA in two other species, *H. megacephalus* (2/201) and *Oligoryzomys mattogrossae* (1/30), which may be due to spillover as these rodents are not known to carry hantaviruses in the MRBR. Although we did not analyze a serum sample from each unique capture, the number of analyzed samples per grid correlated with the estimated rodent abundance per grid session (Figure S3).

First, we established that there was no linear association between total rodent captures and seropositivity (i.e., seroprevalence) (*β_1_* = 0.02, *p* = 0.160, R^2^_adj_ = 0.11), nor was there a linear association between seropositive *A. montensis* (*β_1_* = 0.03, *p* = 0.152, R^2^_adj_ = 0.11) or *O. nigripes* (*β_1_* = 0.04, *p* = 0.268, R^2^_adj_ = 0.03) and their respective per-grid abundances (Figure 3C). However, there was a strong linear association between the density of antibody-positive rodents per grid (estimated by minimum number of antibody positive, “MNAP”) and proportion of antibody-positive rodents per grid (i.e., seroprevalence) for all rodents (*β*_1_ = 0.012, *p* < 0.001, R^2^_adj_ = 0.50), *A. montensis* (*β*_1_ = 0.03, *p* < 0.001, R^2^_adj_ = 0.68), and *O. nigripes* (*β*_1_ = 0.11, *p* = 0.016, R^2^_adj_ = 0.28) (Figure S4). Due to the relatively small number of seropositive animals, we did not attempt to estimate the density of antibody-positive rodents by mark–recapture methods. We therefore elected to use seroprevalence as the response variable (rather than MNAP) to analyze our data by logistic regression as there was a strong linear association between seroprevalence and MNAP with relatively good correlation, and seroprevalence as a binary response allowed us to analyze serostatus at the level of the individual.

For *A. montensis*, age, weight, reproductive activity, and the presence of a tail scar (indicative of *Leishmania* sp. infection) were all associated with the presence of antibodies to hantavirus, whereas for *O. nigripes*, the factors sex, age, reproductive activity, and weight were associated with the presence of antibodies to hantavirus (Table 3). Specifically, seropositive *A. montensis* were heavier adults with a tail scar: the odds of adults being seropositive were 3.23 times higher than sub-adults (*p* = 0.022); reproductively active individuals were 3.35 times more likely to be seropositive (*p* = 0.004); the odds of an individual with a tail scar having hantaviral antibodies were 2.75 times higher than those without a tail scar (*p* < 0.019); and the mean weight of seropositive individuals was 36.4 g, whereas the mean weight of seronegative individuals was 23.4 g (odds increase 1.08 times for each 1-g increase in weight, *p* < 0.001). As these factors were highly correlated (e.g., adult *A. montensis* individuals were heavier and 10.9 times more likely to have tail scars), multivariable tests were not performed.

*Oligoryzomys nigripes* that were seropositive for hantavirus were heavier, reproductively active males: the odds of being antibody-positive were 11.02 times higher for males than females (*p* = 0.023), reproductively active individuals were 7.08 times more likely to be seropositive (*p* = 0.013), and the odds of seropositivity increased 1.20 times for each 1-g increase in weight (*p* = 0.004) (Table 3). Age was a perfect predictor of being seropositive for hantavirus in *O. nigripes*, as only adults (14 of 99 tested) were positive for hantaviral antibody; all sub-adults (*n* = 23) and juveniles (*n* = 1) were seronegative. In all cases, post hoc power analysis showed that sample sizes of these two species were sufficient to detect statistical associations (1-β > 0.90).

### 3.5. Relationship between Species Diversity and Hantavirus Prevalence

As we determined that we indirectly altered species diversity by adding resources to certain grids, we tested whether there was a statistical relationship between the Shannon’s index and seropositivity. We found no linear association between Shannon’s index and the proportion of rodents seropositive for hantaviral antibodies (*β*_1_ = −1.15, *p* = 0.641, R^2^_adj_ = −0.05), no association when controlling for repeated measures (*p* = 0.618), and no association when controlling for resource augmentation (*p* = 0.47) (Figure 3B).

### 3.6. Effect of Resource Augmentation and Habitat Degradation on Hantavirus Seroprevalence

We tested whether being hantavirus-seropositive was associated with the experimental variables of resource augmentation and/or habitat degradation (fixed variables), controlling for sampling grid (random variable) using logistic regression.

We found no statistical association with resource augmentation and prevalence of hantavirus in *A. montensis* (*p* = 0.734) or *O. nigripes* (*p* = 0.673) (Table 4). However, we detected a significant association between antibody-positive animals and habitat degradation. Specifically, *A. montensis* captured in habitats with a moderate level of degradation were 3.68 times as likely to be seropositive to hantavirus as those in the least degraded habitats (95% C.I. = 1.33~11.8) while animals in the most degraded habitats were only 1.88 times as likely (95% C.I. = 0.59~6.47, not significantly different than least degraded habitat) (Table 4). Mixed-effect models which included random effects of grid and main effects of resource augmentation or habitat degradation suggested that within-grid variability did not contribute significantly to the variance (not shown). Multivariable models with resource augmentation and habitat degradation did not significantly explain additional variance, suggesting that habitat degradation is an independent predictor of hantaviral antibodies in *A. montensis* (not shown). This confirms previous observations that there is a relationship between hantavirus-seropositive, reservoir rodents and habitat degradation [17,33].

### 3.7. Fine-Scale Association between Rodent Microhabitat and Hantavirus Prevalence

**Figure 4:**
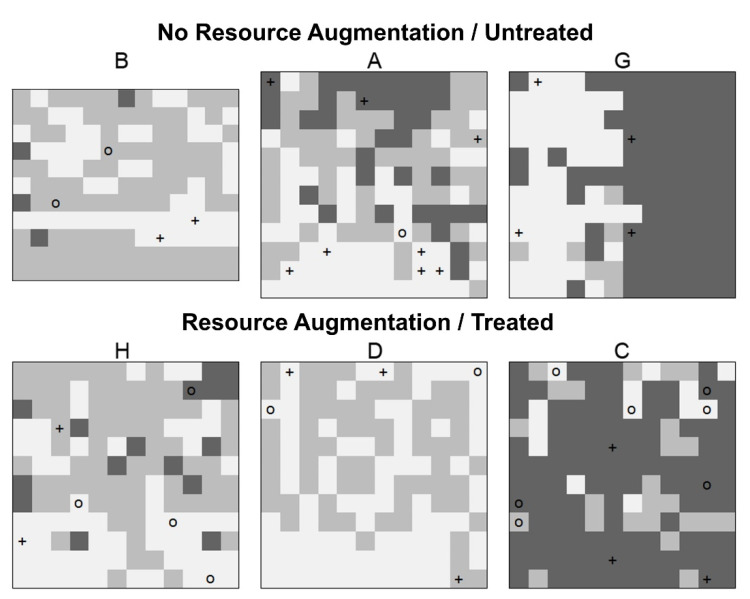
**Hierarchical clustering based on environmental characteristics measured per trapping station.** Hierarchical clustering revealed fine-scale habitat clusters based on environmental characteristics measured per trapping station (Cluster 1, light gray; Cluster 2, gray; Cluster 3, dark gray). The vegetative indices were sampled during June–July (winter, dry season) of 2015. The grids were previously classified by overall habitat degradation (left to right: least degraded, Grids **B** and **H**; moderate degradation, Grids **A** and **D**; and most degraded, Grids **G** and **C**) for untreated grids (top row) and grids with resource augmentation (“Treated”, bottom row, Grids **H**, **D**, and **C**). Circles show the location of antibody-positive *A. montensis*, and “+” show the locations of all other rodent species with hantavirus-reactive antibodies.

As we determined that habitat degradation was an independent predictor of the presence of hantaviral antibodies in *A. montensis*, and not species diversity or resource augmentation, we were interested in defining the microhabitat associations of rodents and hantaviral seroprevalence within the study area. Previously, we showed that hantavirus seroprevalence was associated with habitat as defined by a dominant vegetation type [37]. Initially, we used three broad categories to define the level of habitat degradation (least, moderate, and most degraded habitats), based on grid averages for six vegetation parameters measured at each grid station (Supplemental Text). However, further analysis of station-level vegetation measurements revealed that each grid was heterogeneous (Figure 4; Figure S1). As the data suggested that the distribution of these rodents was associated with fine-scale “microhabitat” characteristics (Table S3) [36], we were interested in whether seropositive mice were also associated with these fine-scale microhabitat types. We therefore employed hierarchical clustering to classify sampling stations into three major clusters according to five of the six vegetation parameters used above to classify degradation level, and each cluster could be defined by specific dominant vegetation characteristics and degradation status (Figure 4; Supplemental Text, Figure S5).

There was no statistical association between *O. nigripes* and fine-scale habitat classification (*χ*^2^ = 1.53, d.f. = 2, *p* = 0.465), or between the microhabitat clusters and virus-reactive antibody positivity in *O. nigripes* (Table 4). However, there was a statistical association between the presence of *A. montensis* and microhabitat cluster (*χ*^2^ = 8.21, d.f. = 2, *p* = 0.016); specifically, the odds of capturing *A. montensis* at a Cluster 2 trap station were 1.64 (95% C.I. 1.17~2.32) times the odds of catching *A. montensis* on Cluster 1 (Table 4, Table S4). Of interest, the odds of capturing an antibody-positive *A. montensis* in Cluster 2 were 0.18 (95% C.I. 0.04~0.58) times that of Cluster 1 (1% of trap sites in Cluster 2 had hantavirus antibody-positive *A. montensis*, compared to 8% in Cluster 1 and 5% in Cluster 3) (Table 4, Table S4). Fine-scale habitat clusters showed that *A. montensis* was generally associated with habitats containing a high percentage of deadwood (Cluster 2); however, more antibody-positive *A. montensis* were identified than expected in habitats with a high percentage of grasses and no fallen trees or orange trees (Cluster 1, least degraded habitats) (Figure S5).

As we determined that *A. montensis* was associated with fine-scale habitat clusters, we then used species distribution models to define the niche and test whether antibody-positive individuals were found within their niche. Species distribution models were based on seven vegetation variables (the six above plus maximum canopy height) and trained using PreTrt capture data (Supplemental Text; Figure S6; Table S3). Post-treatment data and model performance showed that the niche of *A. montensis* (but not *O. nigripes*) could be accurately predicted, and resource augmentation did not change the predicted probability of capturing *A. montensis* at a given station (Supplemental Text; Figures S5 and S7; Tables S5 and S6). Similar to the results of the microhabitat cluster analysis above, the odds of capturing a seropositive *A. montensis* at a predicted capture site were 0.31 (0.10~0.78) times the odds of capturing a seropositive at other sites—thus, seropositive *A. montensis* were unlikely to be found within the species’ fine-scale microhabitat niche.

## 4. Discussion

Conducting field experiments to address a specific hypothesis in a biotically rich environment such as the Atlantic Forest is challenging but necessary to test the significance of findings reported from longitudinal, observational studies of wildlife zoonoses. Recent investigative efforts have focused on the effects of forest fragmentation on small mammal community richness [55], persistence [43], and species diversity and hantavirus prevalence (e.g., [16,56]). Although habitat fragmentation is undoubtedly an important factor in virus–host ecology in the neotropics, the focus of the present study experimentally addressed the role of habitat degradation and microhabitat structure on virus–host ecology, within a non-fragmented forest matrix.

Herein, we tested two factors that have been hypothesized as mechanisms that promote the emergence of hantaviruses harbored by rodent reservoirs within their native ecosystem: (1) food resources and (2) landscape composition. Although resource augmentation changed species community composition (in terms of species diversity and individual species’ population dynamics), it did not affect the prevalence of hantavirus transmission in the population (as measured by prevalence over time), nor was there evidence of a species dilution effect. Further, we observed that the association between habitat and seropositivity was species-specific. Prevalence of hantavirus infection among *A. montensis* was associated with habitat (grid-scale degradation level, as well as fine-scale microhabitat) but there was no association with hantavirus infection and habitat among *O. nigripes*. Additionally, we showed that there was no association between density/movement and resource augmentation. Our findings reflect the complex associations of landscape composition, habitat type, and virus prevalence.

In this study, we experimentally added food resources to one of each of the three pairs of grids and observed changes in the rodent community (Figure 1, Table 1). We observed that the density of individuals increased for two of the three most abundant species, the proportion of individuals captured increased for many species, and the species diversity increased on treated grids (Figure 3). Increased reproduction and increased rodent density have been observed for the tropical species studied here in relation to food availability [57,58,59,60]. The increased prevalence of hantavirus in rodent reservoirs has been linked to cycles of increased resources in natural neotropical forests, specifically due to the increased population density of reservoir rodents [61]. However, we detected no association between hantavirus prevalence and resource augmentation. One limitation of our study design is that we were unable to assess the independent relationship of these effects with hantavirus seroprevalence, as density, abundance, and species diversity are likely correlated. It is therefore possible that one effect is masking another with respect to seroprevalence. However, an earlier study by our group found no correspondence between seroprevalence and population density in *A. montensis*, suggesting that other factors might be more important than simple demographics in maintaining the virus in the reservoir population [34]. Similarly, a recent study reports that hantavirus seroprevalence was not directly influenced by host (*Myodes* and *Apodemus*) abundance in Hungary [62], and a multi-year study found that maternal antibodies may dampen the direct effects of increased rodent density on increased *Puumala orthohantavirus* seroprevalence [26].

Collectively, the data suggest that increased resources may cause immediate increases in the rodent population (density or abundance within one generation) in some reservoir species, which may affect hantavirus prevalence or transmission, but the affect is species-specific within the same habitat. Extended to our broader hypothesis, ecological factors that influence hantavirus prevalence in the short term may not be universally applicable to each rodent reservoir or rodent community across various ecosystems or even within the same ecosystem. Longer-term studies are necessary to capture the cyclical dynamics of hantavirus–rodent abundance, and short-term studies such as ours are necessary to guide long-term studies. Our data support the idea that finer-scale analyses (i.e., microhabitat) provide more information about the potential transmission dynamics of rodent-borne hantavirus maintenance and transmission.

The dilution effect is a hypothesis that posits a negative association between the prevalence of a pathogen in a host species population (i.e., highly competent rodent reservoirs) and increased biodiversity of the community [63]. Experimental and observational studies in the Americas demonstrate that the prevalence of some rodent-borne hantaviruses in their respective reservoir host species decreases with the increasing species diversity of the rodent community (e.g., [2,64,65]). However, a recent meta-analysis that examined species diversity in studies reporting the prevalence of hantaviruses in various rodent reservoirs in the Americas found a lack of support for the hypothesis; therefore, the association between biodiversity and hantavirus prevalence may vary by virus/rodent reservoir system [66]. We indirectly increased rodent species diversity by adding resources to certain grids (Figure 3A); however, we found no evidence of a dilution effect or of an amplification effect (Figure S1).

One plausible biological explanation for the dilution effect with respect to hantavirus transmission is that increased species diversity reduces intraspecific contact among reservoir species, potentially due to behavioral changes which arise from interspecific interactions [3,65]. We previously demonstrated that *A. montensis*, *O. nigripes*, and the potential spillover species (*H. megacephalus*) have similar habitat preferences [34] but tend to avoid other species on small spatial scales [36]. More importantly, fine-scale conspecific associations were detected independently of habitat; thus, the opportunity for intraspecific transmission is similar across all habitats [36]. Indeed, species distribution models predicted relatively little overlap between the three predominant species (Table S6). The data support the broader notion that the relationship between biodiversity and hantavirus prevalence is complex and dependent on the specific host–virus ecology [66,67]. Thus, factors which affect hantavirus–rodent activity in one ecosystem may not be broadly applicable to others.

Our field experiments conclusively demonstrate that habitat and landscape composition are primary factors for the prevalence of hantavirus in *A. montensis* in the neotropics, but not for *O. nigripes*. In our studies, we used two approaches to assess habitat: (1) a landscape degradation classification based on grid average, referred to as habitat degradation, and (2) a station-scale “microhabitat” classification based on hierarchical clustering, referred to as station clusters. There are clear advantages of analyzing landscape effects at more than one hierarchical level [38]. Muylaert et al. (2019) reported that the proportion of a hantavirus reservoir in their Atlantic Forest site showed a higher positive correlation with landscape diversity than by a particular habitat matrix [32]. In our study, there was an increased likelihood of detecting an antibody-positive *A. montensis* on grids with moderate habitat degradation, but on a fine scale, we detected fewer seropositive *A. montensis* at stations where fallen trees were present (Cluster 2) and no difference between stations with non-native orange trees (Cluster 3, i.e., most degraded habitats) and stations with a higher percentage of grasses where orange trees were absent (Cluster 1). However, habitat did not influence the probability of hantavirus infection of *O. nigripes*, at either the grid scale or the station scale.

*Akodon montensis* is often the most abundant small mammal encountered in field studies in the Atlantic Forest of Southern Brazil, Northeastern Argentina, and Eastern Paraguay. It is considered a habitat generalist, although several studies have demonstrated associations with a variety of particular environmental characteristics such as terrestrial ferns and trunks [68], high abundance of bamboo [44], or open canopy [69]. Recent work by our research group has described the non-monotonic association of *A. montensis* population density with habitat degradation [36,40,70], with the largest populations encountered in moderately degraded habitat and fewer *A. montensis* in both least and most degraded habitats. Both the grid-level and station-level results of the present study further support this finding—the grids with moderate degradation and the Cluster 2 stations showed higher population levels or occupation rates for *A. montensis*.

*Oligoryzomys nigripes* is another commonly encountered rodent in the Atlantic Forest and was the third most common species in our study. In comparison with *A. montensis*, *O. nigripes* is reported to be more specialized in its habitat requirements, being variously reported to prefer areas “associated to a low canopy and a dense understory” [69] or with “high density of scrubs and negatively correlated to mature forest indicators” [44]. However, a recent study by our research group found that *O. nigripes* did not exhibit an association with any level of habitat degradation [65], and the results of the present study extend this finding to a microhabitat level, where there was no difference in the association of *O. nigripes* with any of the station clusters and only weak support for a species distribution model based on station-level vegetation characteristics.

Further, we reported that *A. montensis* was more likely to be antibody- and/or RNA-positive for hantavirus in “High Forest” habitat [37] and specifically was associated with microhabitat with greater overstory [39]. In summary, these reports demonstrated that habitat type exerts a strong influence on viral maintenance in *A. montensis* in the Atlantic Forest. In the present study, although *A. montensis* exhibited higher population density in the moderately degraded habitats and higher occupation rates of the Cluster 2 stations, we found a negative association of viral seroprevalence with Cluster 2 stations. Furthermore, species distribution models showed that seropositive *A. montensis* were not captured in predicted microhabitats (i.e., predicted based on species distribution models), although the models otherwise performed well with predicting the presence of seronegative *A. montensis* at a specific station. Again, lower seroprevalence associated with higher population levels suggests that the hypothesized dilution effect is not ubiquitous and may be of less importance in an ecosystem of high plant and animal diversity such as the Atlantic Forest. In contrast to our findings for *A. montensis*, habitat did not influence the probability of hantavirus infection of *O. nigripes*, at either the grid scale or the station scale.

Our previous studies and those of other researchers have shown that land use conversion may drive systematic increases in rodent-borne pathogen prevalence and that this effect may be shown across a variety of rodent reservoirs (e.g., [17,71]). Our field experiments conclusively demonstrate the role of landscape and microhabitat composition as a primary factor in both population composition and hantaviral seroprevalence in the Atlantic Forest for *A. montensis*, the primary reservoir of *Jabora orthohantavirus* in the Paraguayan Atlantic Forest [35,41]. However, for *O. nigripes*, a reservoir for *Juquitiba orthohantavirus*, landscape was not a significant factor for either rodent population density or viral prevalence. An important conclusion to be drawn from this is that the virus–host–environment ecology, and thus the transmission risk and epidemiology, for one strain of hantavirus is not necessarily generalizable to other strains.

## Figures and Tables

**Table 1:** Summary of unique male and female rodents by species collected over the course of the study per session.

	PreTrt		ND2015		FM2016		
Species	Males	Females	Males	Females	Males	Females	Total
***Akodon montensis ^a^***	148	128	117	131	107	91	722
***Calomys callosus***	1	3	4	3	6	4	21
***Calomys tener***	0	0	0	2	0	0	2
***Euryoryzomys russatus ^b^***	0	0	7	1	0	0	8
***Holochilus chacarius***	0	0	1	0	1	0	2
***Hylaeamys megacephalus ^c^***	46	30	44	36	67	47	270
***Nectomys squamipes***	0	0	0	1	0	1	2
***Oligoryzomys flavescens***	0	0	1	4	14	7	26
***Oligoryzomys mattogrossae***	4	6	10	6	14	6	46
***Oligoryzomys nigripes***	23	15	33	28	34	21	154
***Rattus rattus***	0	1	0	0	0	0	1
***Sooretamys angouya***	0	0	1	0	0	1	2
**TOTAL**	**222**	**183**	**218**	**212**	**243**	**178**	**1256**

The sampling sessions included one pre-treatment session from June to July 2015 (PreTrt) and two sessions after the addition of resources (post-treatment) in November–December 2015 (ND2015) and February–March 2016 (FM2016). Sex was not determined from: ^a^ 8 individual *A. montensis* from ND2015; ^b^ 1 individual *E. russatus* from ND2015; ^c^ 1 individual *H. megacephalus* from ND2015, and 1 individual *H. megacephalus* from FM2016.

**Table 2:** Percentage of rodents seropositive (“Sero+”) for antibodies to hantavirus by immunofluorescence assay during study by session and by species.

Variable	Level	Sero+/Tested ^b^	% Sero+ ^c^
By Session ^a^	PreTrt	14/276	5.07%
	ND2015	12/266	4.51%
	FM2016	15/402	3.73%
By Species	*Akodon montensis*	24/527	4.39%
	*Oligoryzomys nigripes*	14/123	11.38%
	Other rodents	3/294	1.02%
	Total captured	41/944	4.34%

^a^. Pre-treatment (PreTrt) sampling was performed May–July 2015, first post-treatment mark–release–recapture (MRR) session was performed November–December 2015 (ND2015), second post-treatment capture session was performed February–March 2016 (FM2016). ^b^ Antibody-positive individuals (Sero+)/total tested; ^c^ percentage of unique individuals tested per session.

**Table 3:** Univariate tests of statistical association between the presence of hantavirus-reactive antibodies and rodent characteristics for two species of sigmodontine rodents.

Species	Predictors	Factor Level	No. Neg (%)	No. Pos (%)	Odds Ratio ^a^	*p* ^b^	LR *p*-Value ^b^
*Akodon montensis*	Sex	Male	252 (94)	16 (6)	1.98 (0.8~4.9)	0.123	0.113
		Female	249 (97)	8 (3)			
	Age	Adult	263 (94)	18 (6)	3.23 (1.3~9.9)	0.022	0.021
		Juvenile	4 (80)	1 (20)	11.80 (0.5~101.0)	0.041	
		SubAdult	236 (98)	5 (2)			
	Weight ^c^		501 (9~57)	24 (11~74)	1.08 (1.0~1.1)	0.000	0.000
	Tail Scar	Y	190 (93)	15 (7)	2.75 (1.2~6.6)	0.019	0.017
		N	313 (97)	9 (3)			
	Reprod. Activity	Active	101 (90)	11 (10)	3.35 (1.4~7.7)	0.004	0.006
		Not active	402 (97)	13 (3)			
*Oligoryzomys nigripes*	Sex	Male	59 (82)	13 (18)	11.02 (2.1~203.6)	0.023	0.002
		Female	50 (98)	1 (2)			
	Age ^d^	SubAdult	23 (100)	0			
		Juvenile	1 (100)	0			
		Adult	85 (86)	14 (14)			
	Weight ^c^		109 (6~37)	14 (22~31)	1.20 (1.1~1.4)	0.004	0.001
	Tail Scar	Y	3 (60)	2 (40)	5.89 (0.7~39.1)	0.065	0.091
		N	106 (90)	12 (10)			
	Reprod. Activity	Active	50 (81)	12 (19)	7.08 (1.8~46.9)	0.013	0.003
		Not active	59 (97)	2 (3)			

^a^ Odds ratios for factor levels are in reference to the last level listed and include the parameter estimate followed by the 95% confidence interval in parentheses; ^b^ *p*-values test the statistical significance of the odds ratio and likelihood ratio (LR) test statistics, respectively; ^c^ weight ranges in grams are listed following the number of seronegative/seropositive animals; ^d^ age was a perfect predictor of seropositivity in *O. nigripes*.

**Table 4:** Univariate tests of statistical association between the presence of hantavirus-reactive antibodies in two rodent species and experimental predictors of resource augmentation and habitat degradation classification using logistic regression.

Species	Predictors	Factor Level	No. Neg (%)	No. Pos (%)	Odds Ratio ^a^	*p* ^b^	LR *p*-Value ^b^
*Akodon montensis*	Resource	Y	131 (95)	7 (5)	1.17 (0.44~2.78)	0.734	0.767
		N	372 (96)	17 (4)			
	Habitat Degradation	Most	156 (96)	7 (4)	1.88 (0.59~6.47)	0.287	0.040
		Moderate	137 (92)	12 (8)	3.68 (1.33~11.8)	0.016	
		Least	210 (98)	5 (2)			
	Habitat Clusters	3	139 (95)	7 (5)	0.60 (0.22~1.55)	0.127	0.016
		2	200 (99)	3 (1)	0.18 (0.04~0.58)	0.004	
		1	144 (92)	12 (8)			
*Oligoryzomys nigripes*	Resource	Y	61 (90)	7 (10)	0.79 (0.25~2.44)	0.673	0.673
		N	48 (87)	7 (13)			
	Habitat Degradation	Most	38 (88)	5 (12)	1.05 (0.28~3.75)	0.936	0.996
		Moderate	23 (88)	3 (12)	1.04 (0.20~4.34)	0.955	
		Least	48 (89)	6 (11)			
	Habitat Clusters	3	35 (92)	3 (8)	0.42 (0.09~1.57)	0.223	0.340
		2	32 (91)	3 (9)	0.46 (0.09~1.73)	0.275	
		1	39 (83)	8 (17)			

^a^ Odds ratios for factor levels are in reference to the last level listed and include the parameter estimate followed by the 95% confidence interval in parentheses; ^b^ *p*-values test the statistical significance of the odds ratio and likelihood ratio (LR) test statistics, respectively.

## Data Availability

The data presented in this study are available on request from the corresponding author.

## Supplementary Materials

The following are available online at https://www.mdpi.com/1999-4915/13/1/85/s1, Supplemental Methods description. Figure S1: Habitat vegetation measurements from each trap station used to generate habitat degradation classification by principal components analysis, showing boxplots by grid.; Figure S2. Rodent community structure using hierarchical clustering of Jaccard’s dissimilarity index based on Ward’s distances. Figure S3. Sub-samples of rodents for testing hantavirus-specific antibodies were correlated with rodent abundance. Figure S4. Seroprevalence is highly correlated with density of seropositive rodents. Figure S5. Hierarchical clustering dendrogram based on Gower’s distance using five habitat variables measured at each of 863 trap stations across six sampling grids. Figure S6. Principal component analysis of seven habitat variables measured at each trap station. Figure S7. Rodent distribution probabilities on untreated versus treated grids. Table S1. Summary of total captures (unique individuals) of rodent species captured per grid over the course of the study and species richness per grid. Table S2. Testing the effect of resource augmentation on rodent density and movement based on mark–recapture for six grids over three trapping sessions per grid using spatially explicit capture–recapture models. Table S3. Habitat variables as predictors for the presence of three rodent species at individual trap stations using univariate logistic regression. Table S4. Relationship between fine-scale habitat clusters, broad-scale habitat classification (count of trap stations), and hantavirus reservoirs (counts per trap station). Table S5. Model fitness statistics for species distribution models using logistic regression pre- and post-treatment (training and testing data, respectively); and on untreated control grids and treated (resource augmented) grids during post-treatment sampling sessions. Table S6. The performance of GLM models to predict the presence of sigmodontine species according to environmental variables taken at specific trap sites.
