# Supplementary material for: Mixed Effects of Habitat Degradation and Resources on Hantaviruses in Sympatric Wild Rodent Reservoirs within a Neotropical Forest
Source: Viruses. 2021 Jan 9;13(1):85. doi: 10.3390/v13010085 (PMC7827808; doi:10.3390/v13010085)
Supplement: Supplementary file 1 [file viruses-13-00085-s001.pdf]

Supplementary Information Text

Resource augmentation

Several individuals were captured of each of the two target species (*Akodon montensis, Oligoryzomys nigripes*), for use in food preference trials, to determine the palatability of commercially available foods to be used in the resource augmentation grids. These mice were weighed, housed in separate cages, provided water *ad lib*, and ample amounts of trios of foods were presented during Days 2, 4, and 6 of captivity. The mice were weighed again on Day 7, and the food combinations were ranked in terms of relative weight gain (or loss) for the mice consuming them, with the assumption being that weight gain indicates a preferred and nutritionally appropriate food (or combination of foods). Beginning with eight possible food selections (peanuts, oats, flax, sesame, canary grass, sunflower, and two pet-feed mixtures), by process of eliminatory trials, a preferred food was selected separately for the feeding stations, a commercial pet-food mixture available from "Foody" company in San Lorenzo, Paraguay. The mixture is named "Super Mix", consisting of whole kernel corn, peanuts in the shell, sunflower seeds, rice and a compressed pellet of undisclosed ingredients. This mixture was briefly heated to prevent germination of any seeds, in order to prevent introduction of non-native plant species into the forest.

Grid Selection

Areas across the Mbaracayú Forest Biosphere Reserve (MFBR) were sampled previously for rodents and the presence of antibodies to hantavirus at 21 sites [1, 2]. Grid sites for the present study were centered on lines where seropositive animals had been encountered in the earlier study. For each of the six grids, habitat variables (vegetation and related structures) were measured at each trapping station to evaluate which grids would be paired as a control and treated grid. Six variables were included in the analyses, as being important descriptors of both forest quality and rodent habitat:

maximum canopy height, minimum distance to nearest trees, percentage coverage by forbs or grasses, logs (fallen trees) in vicinity, and presence of orange trees (*Citrus aurantium*, an introduced species which has acclimatized to disturbed forests (**Fig. S1**). We evaluated sites using both SAHN clustering and Principal Component Analysis with Minimum Spanning Tree (MST). The six sites formed a continuum on a non-branching minimum spanning tree, simplifying the designation of grid pairs (**data not shown**). The three experimental grids were selected from the grid pairs such that they were as nearly equidistant from each other as possible on the MST. Each grid consisted of 12 x 12 trap stations (11 x 13 in one case), with stations 10 m apart, where two Sherman live traps were set on the ground and one 2-3 m above ground in vines or branches, for a total of 288 traps on the ground and 144 above. In the first pre-treatment (PreTrt) session each station had one trap above ground and one on the ground.

Fine-scale habitat classification

To obtain fine-scale habitat classification at each trap station, we calculated the dissimilarity between individual sampling stations based on five environmental variables (percent ground cover by forbs, grasses, and deadwood, and two binary variables measuring the presence of fallen trees or orange trees) using Gower's distance ("vegan" package in R) (**Fig S5**). The sampling stations were clustered using the average distances to define three groups and these were compared to the previous grid-based habitat classifications. Two of the microhabitat clusters (Cluster 1 and Cluster 2) were divided between the least degraded and moderate degradation categories (**Table S4**). Cluster 1 was characterized by a high percentage of grasses and no fallen trees or orange trees; and Cluster 2 was characterized by the presence of fallen trees and a high percentage of deadwood (**Fig. S5**). The third group (Cluster 3) was most similar to the previous highly degraded habitat category, with 67% of trap stations in highly degraded

habitat grids characterized by the presence of orange trees and a significantly higher percentage of forb ground cover (**Fig. S5**). However, this microhabitat cluster was also found on grids previously characterized as least degraded (7% of trap stations) or moderate degradation habitats (12% of trap stations) (**Table S4**). Thus, although easily classified by their general levels of habitat degradation, each grid was also heterogeneous when evaluated on a station-scale (**Fig. 4**). Our analyses of rodent species and hantavirus distribution on two scales (grid- and station-) facilitated two distinct approaches, enabling us to evaluate two distinct biological questions. Grid-scale analysis evaluates the effect of the habitat matrix on the rodent community and populations and hantavirus antibody seroprevalence, whereas station-scale analysis provides insight into the individual rodents' microhabitat preferences, enabling evaluation of each species, sex, age-group and/or serostatus separately.

Prediction of habitat association

There were clear associations between the specific vegetation features and the presence of a given rodent species (**Table S3**). As we also detected an association of hantavirus antibody-positive *A. montensis* with specific microhabitat types, but not *O. nigripes* (**Table 4**), we used species distribution modeling to predict the presence of the rodent species. We followed published guidelines for reporting ecological niche modeling and species distribution modeling [3]. Pre-treatment grids were used to train a logistic regression model for each species (*A. montensis*, *H. megacephalus*, and *O. nigripes*), where presence/absence was recorded for each species per trap station over 5 trap-nights per session. The model used principal components for seven vegetation variables to predict species distributions per trap station (**Fig. S6**). In addition to the six variables used in the habitat degradation classification, the percent ground cover by deadwood was included, as it was determined to have high variable loading in a principal

components analysis and was associated with the capture frequency of some species (**Fig S1**; **Fig S6**; **Table S3**). Thus, the resolution of the environmental variables matched the resolution of the presence and absence data, and each trap station was considered an independent sampling unit regardless of grid. Therefore, we did not account for spatial autocorrelation. In general training models performed well, with specificity >0.84, overall accuracy >0.67, and a relatively high TSS (range 0.24~0.31) for each species (**Tables S5 and S6**).

We tested whether the models predicted species captures on post-treatment grids (i.e., if individuals were captured in similar locations over the course of the study). The model accurately predicted the location of *A. montensis* at post-treatment capture sites across all grids (T-test=0.675, p = 0.500) and the model performance statistics reflected this (**Table S6, Fig. S7**). However, the models did not accurately predict the observed captures of *O. nigripes* (T-test = 3.41, p = 0.001) or *H. megacephalus* (T-test = 2.85, p = 0.005) on post-treatment grids (i.e., testing data), and the model performance statistics showed a poorer predictive power over all grids post-treatment (**Table S5**). We then tested whether this poor model performance was due to resource augmentation. There was no difference between the predicted probability of *A. montensis* (P_{Am} = 0.44; T-test p-value = 0.229) or of *O. nigripes* (P_{On} = 0.04; T-test p-value =0.054) between experimental treatments on post-treatment grids (**Table S5**). The addition of resources changed the predicted distribution of *H. megacephalus* ($P_{\mathrm{Hm,control}}$ = 0.11; $P_{\mathrm{Hm,treated}}$=0.096; T-test = 6.23; p < 0.001) resulting in a poorer model fit; however the predicted probability of capturing *H. megacephalus* on control grids ($P_{\mathrm{Hm,Control}}$ = 0.12) was the same as on pre-treatment grids ($P_{\mathrm{Hm,Pre}}$ = 0.11; T-test = 1.24; p-value = 0.216) (**Table S5**). In sum, we could not detect an effect of experimental treatment on the predicted probability of observing *A. montensis*, however the predicted probability of observing *H. megacephalus* at a given station was different following resource

augmentation, and the habitat-based model was a poor predictor of *O. nigripes* during post-treatment capture sessions.

We tested whether seropositive *A. montensis* and *O. nigripes* were found within the predicted sites for these species. Similar to the results of the microhabitat clustering approach, the odds of capturing a seropositive *A. montensis* at a predicted capture site were 0.31 (0.10~0.78) times the odds of capturing a seropositive at other sites (**Table S4**). For *O. nigripes* the predicted probability of capturing a seropositive rodent was the same as capturing a seronegative rodent, and as mentioned previously, there was an effect of treatment but no interaction between seropositivity and treatment which would explain the variance in the predicted probability of capturing *O. nigripes* at a given site (**Table S4**)

Of the three other seropositive individuals (two *H. megacephalus* and one *O. mattogrossae*), which are potential spillover infections, all were captured during the second post-treatment sampling session on the same resource-augmented grid. Each was the single capture incidence of these individuals. Only one of the seropositive *H. megacephalus* was found in a capture site predicted to be used by *H. megacephalus*, and none of the three potential spillovers were captured in a site predicted to be used by *A. montensis* or *O. nigripes*. Although the these potential spillover infections were not captured at stations where previously seropositive rodents were captured, reservoir species were also captured at these same stations: one seropositive *H. megacephalus* was captured at the same station at which one *O. nigripes* was captured 3 nights prior, and the other seropositive *H. megacephalus* was captured at a trap station in which one *A. montensis* and one *H. megacephalus* were captured within two nights prior. The seropositive *O. mattogrossae* was captured in a trap in which a *H. megacephalus* was captured the previous night.

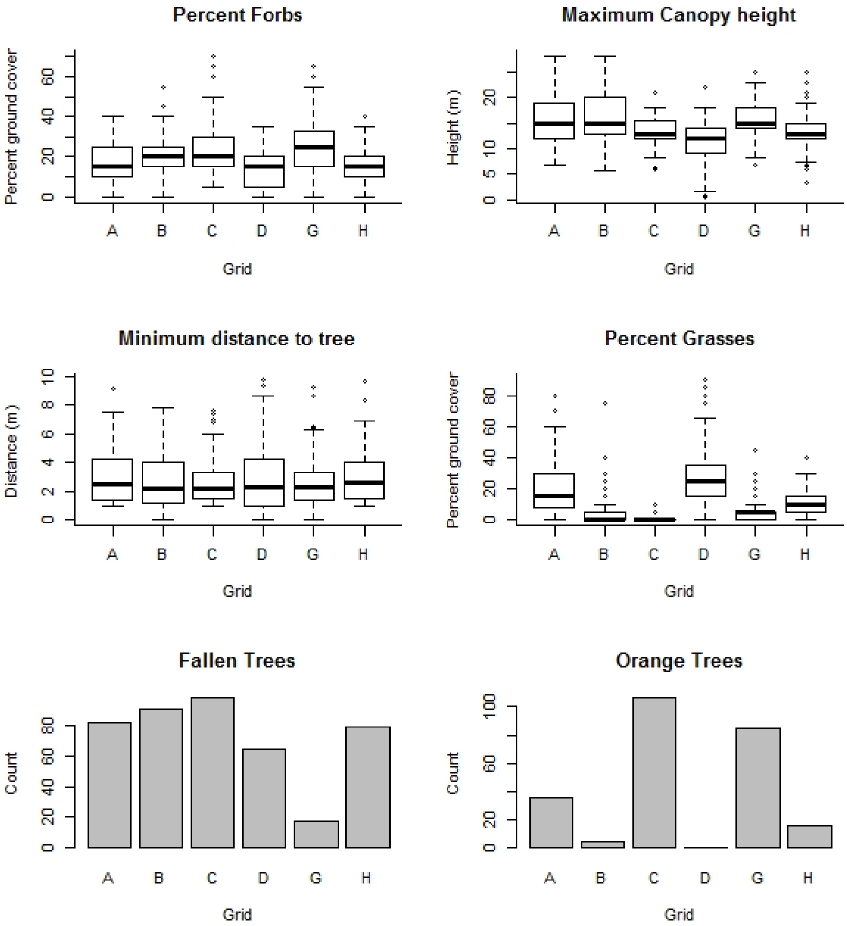

Figure S1. Habitat vegetation measurements from each trap station used to generate habitat degradation classification by principal components analysis, showing boxplots by grid. Four continuous variables are shown (percent ground cover by Forbs or Grasses, Maximum canopy height, and minimum distance to tree) and two binary variables (presence of Fallen trees and presence of orange trees) at each of 863 trap stations across 6 sampling grids. Grids B and H were classified as “least degraded”, grids A and D were classified as “moderate degradation”, and grids C and G were classified as “most degraded” habitats. Additionally, the percent ground cover by fallen trees was included with the above six parameters in a principal components analysis that was used in logistic regression to generate species distribution models of the three

most abundant rodent species; and all variables except minimum distance to trees were used to generate per-station habitat clusters using hierarchical clustering.

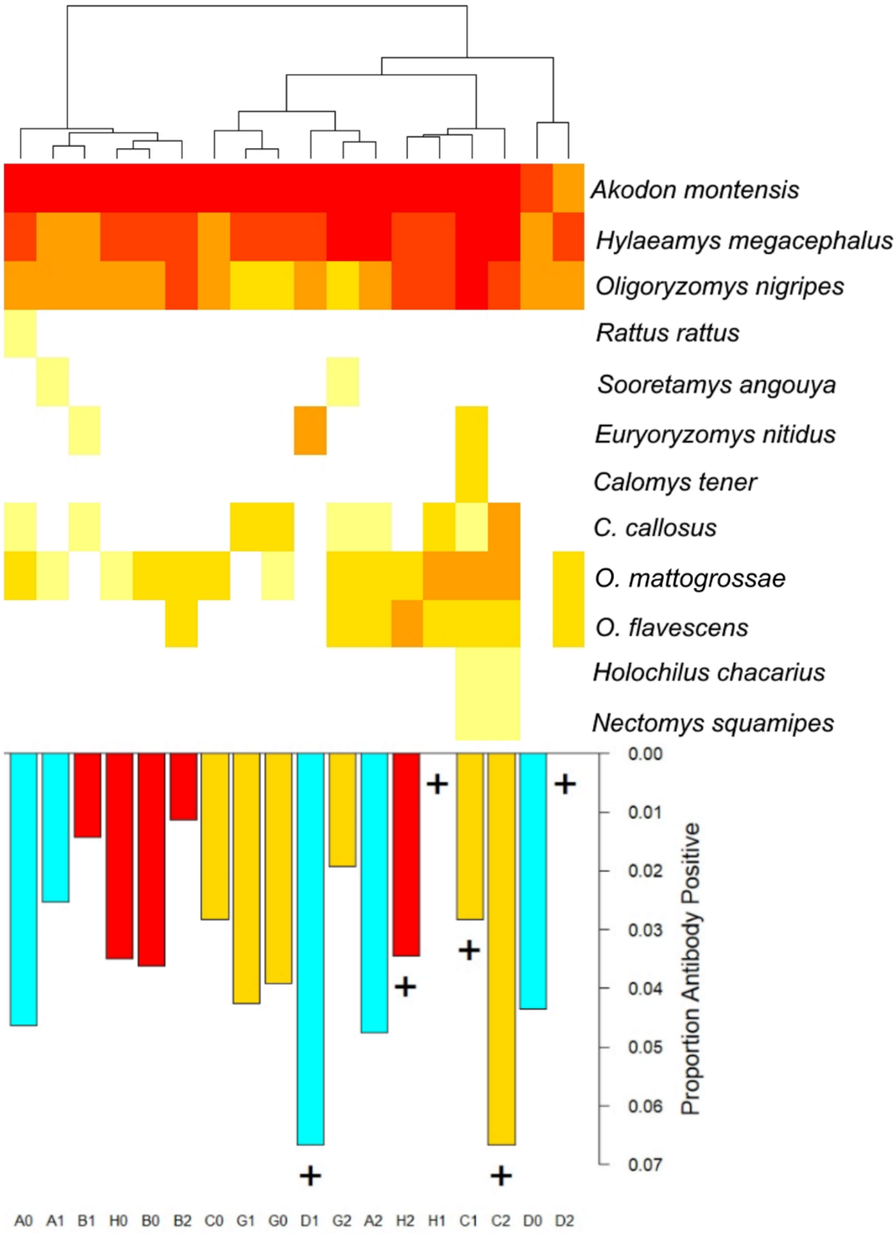

Figure S2. Rodent community structure using hierarchical clustering of Jaccard's dissimilarity index based on Ward's distances. The top portion of the figure (heatmap) shows the scaled relative abundance per grid-session for each species. The bottom portion of the figure shows the proportion of rodents with hantavirus-reactive antibodies ("seropositive") per grid-session. Bars are shaded according to habitat: Grids B and H

were the least disturbed sections of the MFBR (red), moderately disturbed habitats were found in grids A and D (aqua/blue), and mostly disturbed habitats were found in girds C and G (yellow/gold). Grids treated with resources are shown by a “+”. Alphanumeric grid designations under the bars refer to grid letter, and numbers refer to the sampling session (Pre-treatment = 0, ND2014 = 1, FM2015 = 2), e.g., Grid H, 1st post-treatment session = “H1” and Grid D, 2nd post-treatment session = “D2” where no seropositive animals were captured.

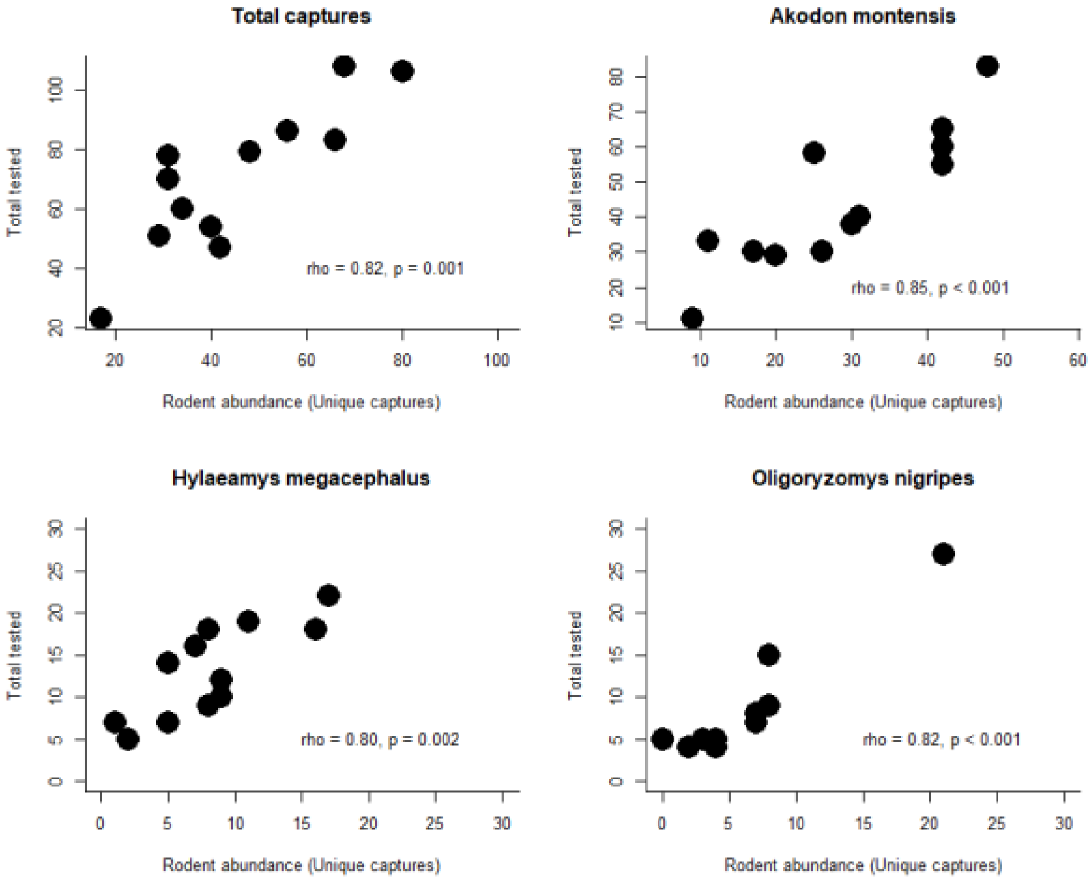

Figure S3. Sub-samples of rodents for testing hantavirus-specific antibodies were correlated with rodent abundance. We analyzed rodent sera for antibodies specific to hantavirus; however not all captured rodents were available for analysis. The number of tested animals (y-axes) significantly correlated with the estimated rodent abundance (unique captures, x-axes) for all species (top left) as well as the three most frequently captured species: the two hantavirus reservoirs, *Akodon montensis* (top right) and *Oligoryzomys nigripes* (bottom right), as well as a potential spillover species, *Hylaeamys megacephalus* (bottom left). Included within each panel are Spearman's rho estimate and *p*-value of the test of significant correlation.

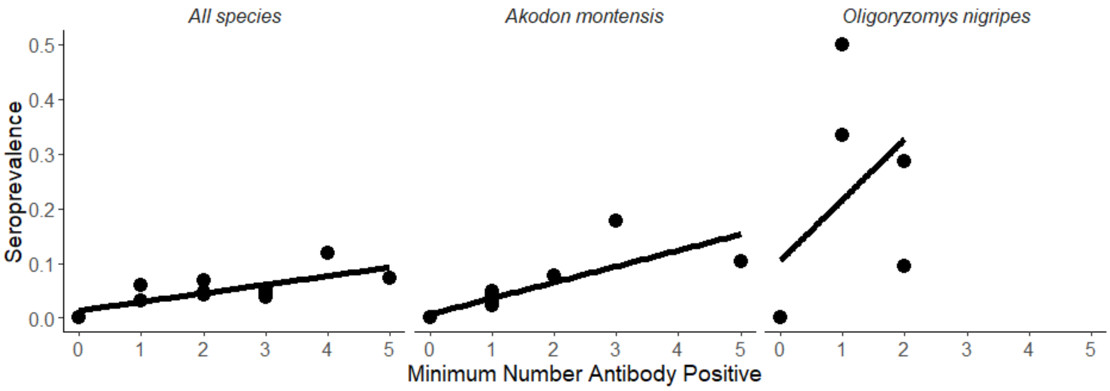

Figure S4. Seroprevalence is highly correlated with density of seropositive rodents. The seroprevalence (i.e., total antibody-positive rodents divided by all analyzed rodents per grid-session) as a function of the minimum number of antibody positive rodents ("MNAP", i.e., density/total seropositive per grid) for all species combined (left), *Akodon montensis* (center) and *Oligoryzomys nigripes* (right). There was a significant linear statistical relationship between seroprevalence and the minimum number of antibody positive rodents per grids-session, disregarding repeated measures and experimental variables. The apparent non-linear relationship between *O. nigripes* seroprevalence and MNAP could not be explained by experimental variables.

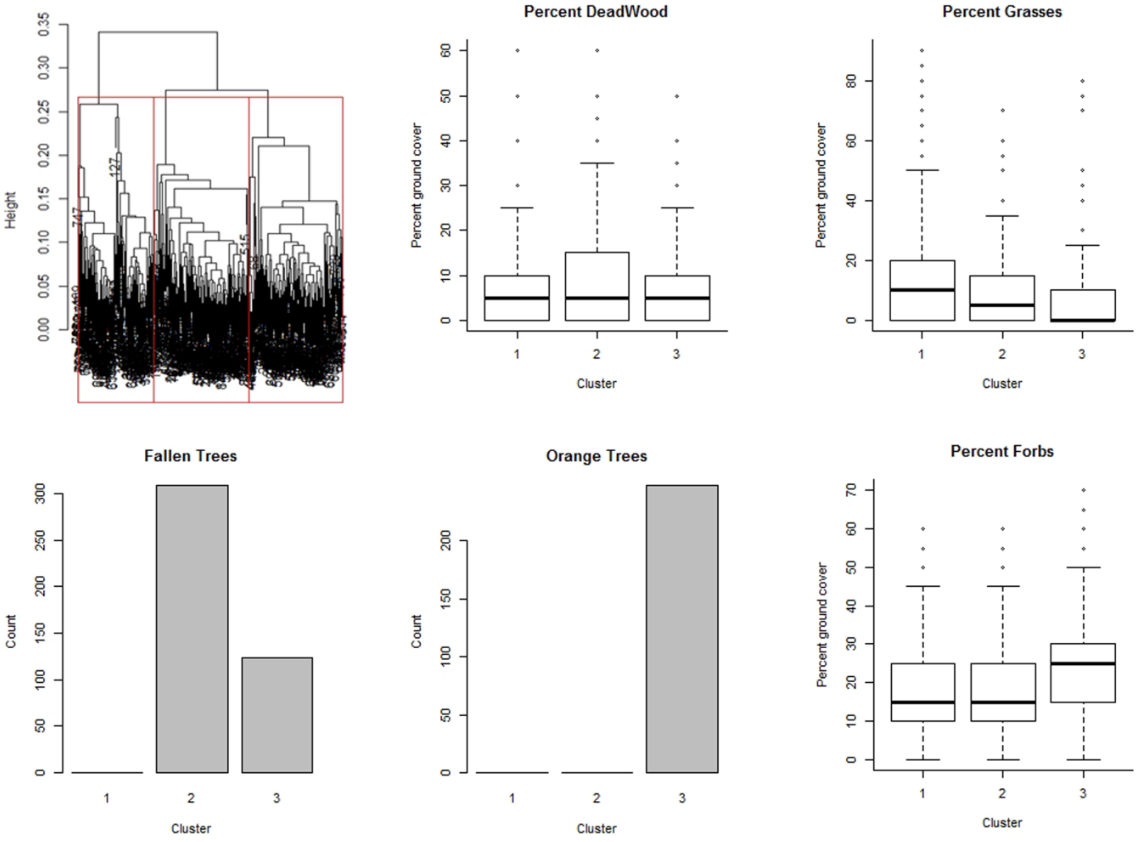

Figure S5. Hierarchical clustering dendrogram (top left) based on Gower's distance using five habitat variables measured at each of 863 trap stations across six sampling grids. Three microhabitat clusters were identified using the 'vegan' package in R based on average distance (red rectangles in top left panel). The remaining panels show the characteristics of trap stations within each cluster based on habitat variables.

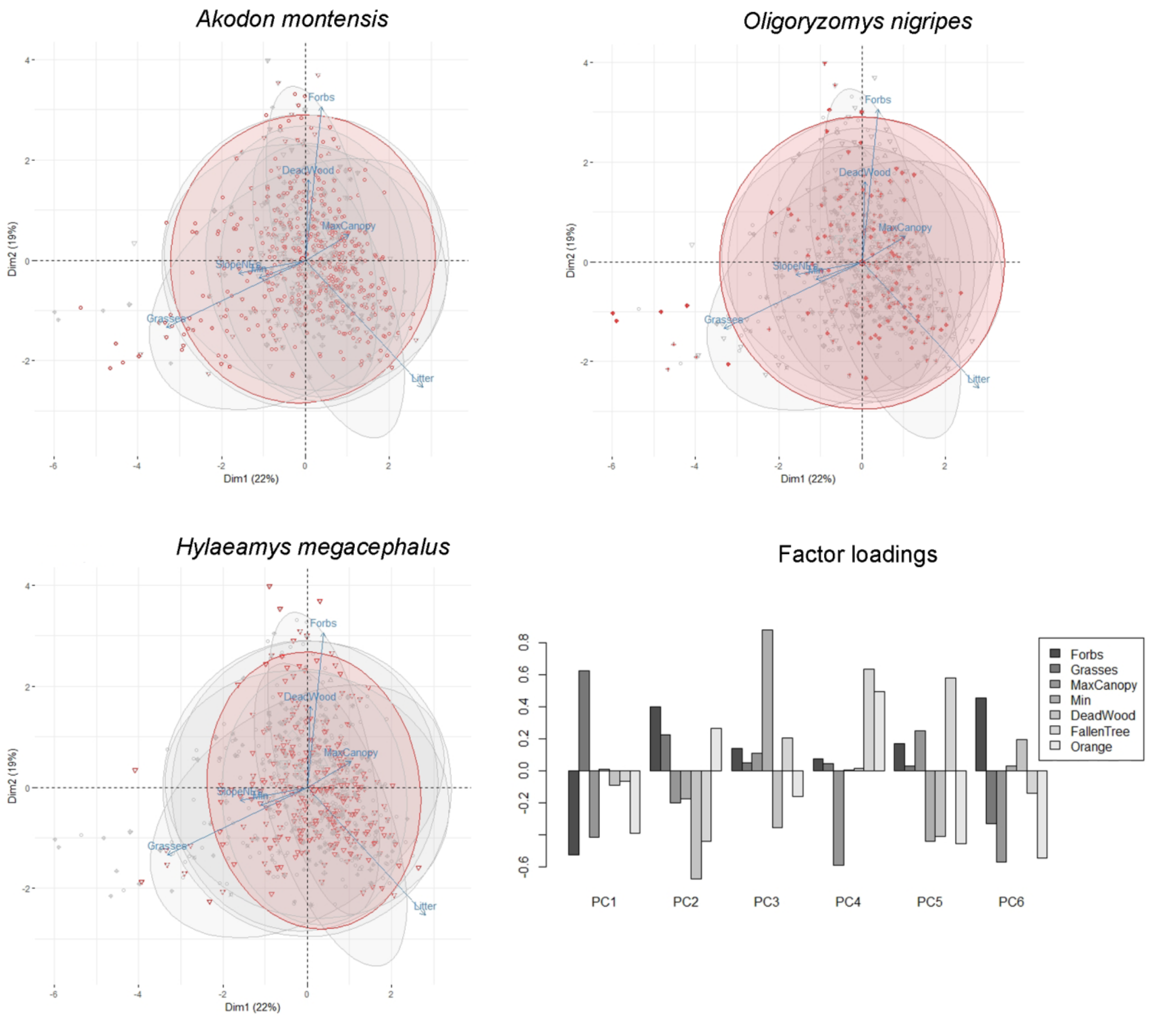

Figure S6. **Principal components analysis of seven habitat variables measured at each trap station**. Colors and red shaded ellipses show sites where each of three species were captured: *Akodon montensis*, *Oligoryzomys nigripes*, and *Hylaeamys megacephalus*. The panel in the bottom right shows the factor loadings for each of the seven habitat variables onto the six principal components. These six components were used to develop station-level species distribution models for the three species shown using generalized linear models.

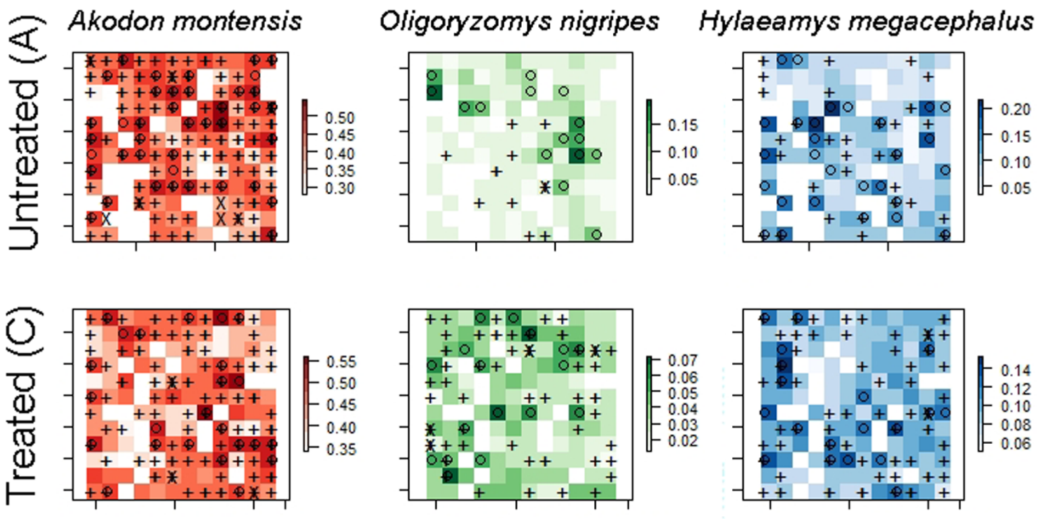

Figure S7. Rodent distribution probabilities on untreated versus treated grids. The predicted probabilities of *Akodon montensis*, *Oligoryzomys nigripes*, and *Hylaeamys megacephalus* on two sampling grids (untreated control grid above, grid treated by augmenting resources below). Predicted probabilities (color scale given below for each species) are based on logistic regression using principal components from seven vegetation parameters measured at each grid-station. Open circles show predicted sites based on habitat characteristics, plus symbols (+) show observed captures post-treatment, and X's show seropositive species on these grids.

Table S1. Summary of total captures (unique individuals) of rodent species captured per grid over the course of the study and species richness per grid.

Grid:	**B**	**A**	**G**	**H**	**D**	**C**	
Resource Augmentation:	**-**	**-**	**-**	**+**	**+**	**+**	
Habitat Degradation	**Least**	**Moderate**	**Most**	**Least**	**Moderate**	**Most**	
Species							Total
Akodon montensis	293 (153)	360 (161)	148 (73)	201 (125)	106 (44)	192 (107)	**1300 (663)**
Calomys callosus	1 (1)	2 (2)	15 (6)	5 (2)	0 (0)	10 (9)	**33 (20)**
Calomys tener	0 (0)	0 (0)	0 (0)	0 (0)	0 (0)	7 (2)	**7 (2)**
Euryoryzomys nitidus	1 (1)	0 (0)	0 (0)	0 (0)	12 (6)	2 (2)	**15 (9)**
Holochilus chacarius	0 (0)	0 (0)	0 (0)	0 (0)	0 (0)	3 (2)	**3 (2)**
Hylaeamys megacephalus	49 (32)	54 (38)	55 (39)	71 (46)	61 (34)	96 (61)	**386 (250)**
Nectomys squamipes	0 (0)	0 (0)	0 (0)	0 (0)	0 (0)	3 (2)	**3 (2)**
Oligoryzomys flavescens	4 (4)	3 (3)	2 (2)	12 (10)	2 (2)	14 (5)	**37 (26)**
Oligoryzomys mattogrossae	9 (5)	8 (8)	3 (3)	21 (12)	4 (4)	18 (12)	**63 (44)**
Oligoryzomys nigripes	28 (20)	26 (19)	13 (10)	62 (39)	26 (15)	71 (41)	**226 (144)**
Rattus rattus	0 (0)	1 (1)	0 (0)	0 (0)	0 (0)	0 (0)	**1 (1)**
Sooretamys angouya	0 (0)	1 (1)	1 (1)	0 (0)	0 (0)	0 (0)	**2 (2)**
TOTAL	**385 (216)**	**455 (233)**	**237 (134)**	**372 (234)**	**211 (105)**	**416 (243)**	***2076 (1165)***
**Species Richness**	**7**	**8**	**7**	**6**	**6**	**10**	**12**

Table S2. Testing the effect of resource augmentation on rodent density and movement based on mark-recapture for six grids over three trapping sessions per grid using spatially explicit capture-recapture models (SECR package in R). A half-normal model was fitted with buffer width of 4-times the movement estimate, allowing both density (*D*, ha^{-1}) and movement (sigma, m) coefficients to vary with treatment (resource augmentation). Model fitness (AIC) is compared to the null model by subtraction (ΔAIC).

**Species**	**Coefficients**	**Untreated Estimate (95% C.I.)**	**Treated Estimate (95% C.I.)**	**AIC**	**ΔAIC**
Akodon montensis	*D*	**62.6 (53.1~73.8)**	**39.8 (32.6~48.5)**	4511.4	8.98
	g0	0.09 (0.07~0.10)	0.09 (0.07~0.10)		
	Sigma	9.75 (9.11~10.4)	10.5 (9.75~11.3)		
Hylaeamys megacephalus	*D*	**7.5 (4.69~12.1)**	**24.0 (18.0~32.1)**	1503.2	14.2
	g0	0.02 (0.02~0.03)	0.02 (0.02~0.03)		
	Sigma	21.0 (16.7~26.3)	16.4 (14.1~19.1)		
Oligoryzomys nigripes	*D*	**7.0 (3.75~13.1)**	**18.4 (13.6~25.0)**	1134.6	7.2
	g0	0.04 (0.03~0.06)	0.04 (0.03~0.06)		
	Sigma	13.4 (9.98~18.0)	13.5 (11.6~15.7)		

Table S3. Habitat variables as predictors for the presence of three rodent species at individual trap-stations using univariate logistic regression. The presence of a rodent at 863 individual trap-stations on 6 trapping grids were tested for statistical association with linear predictors (percent ground cover by Forbs, Grasses, or Deadwood; Maximum Canopy height, minimum distance to nearest tree ["Tree Dist."]) and two binary categorical predictors (presence of fallen trees; presence of Orange trees) within a 2 m radius of the trap station. Odds ratios are given with 95% confidence intervals, the generalized linear model is compared to the null model for fitness using the likelihood ratio test, and the p-value from that test is reported ("LR p-value").

Species	Predictors	Odds Ratio	p value	LR p value
Akodon montensis	**Forbs**	**1.01 (1.00~1.02)**	**0.009**	**0.008**
	Grasses	1.00 (0.99~1.01)	0.624	0.624
	**Deadwood**	**1.01 (1.00~1.02)**	**0.031**	**0.030**
	MaxCanopy	1.01 (0.98~1.04)	0.570	0.570
	Tree Dist.	1.03 (0.97~1.10)	0.347	0.347
	**Fallen tree**	**1.48 (1.17~1.88)**	**0.001**	**0.001**
	Orange tree	0.85 (0.65~1.10)	0.225	0.224
Oligoryzomys nigripes	Forbs	0.98 (0.95~1.00)	0.080	0.069
	**Grasses**	**1.02 (1.01~1.03)**	**0.000**	**0.001**
	Deadwood	1.00 (0.97~1.02)	0.844	0.843
	MaxCanopy	0.98 (0.92~1.05)	0.618	0.617
	Tree Dist.	1.13 (0.98~1.29)	0.092	0.101
	Fallen tree	0.91 (0.53~1.58)	0.748	0.748
	Orange tree	0.97 (0.51~1.75)	0.926	0.926
Hylaeamys megacephalus	Forbs	1.00 (0.99~1.02)	0.680	0.681
	**Grasses**	**0.98 (0.97~0.99)**	**0.021**	**0.011**
	Deadwood	1.00 (0.98~1.02)	0.975	0.975
	**MaxCanopy**	**1.07 (1.02~1.11)**	**0.003**	**0.003**
	Tree Dist.	0.96 (0.86~1.07)	0.512	0.509
	Fallen tree	0.89 (0.61~1.31)	0.558	0.559
	Orange tree	0.86 (0.55~1.32)	0.496	0.491

Table S4. **Relationship between fine-scale habitat clusters, broad-scale habitat classification (count of trap stations), and hantavirus reservoirs (counts per trap station).**

Factor	Levels	Cluster 1	Cluster 2	Cluster 3
**Habitat Class**	Least Degraded	109	158	20
	Moderately Degraded	132	120	36
	Most Degraded	65	31	192
Akodon montensis	Absent	114	82	77
	Present	192	227	171
	Seronegative	144	200	139
	Seropositive	12	3	7
Oligoryzomys nigripes	Absent	240	253	194
	Present	66	56	54
	Seronegative	39	32	35
	Seropositive	8	3	3

Table S5. Model fitness statistics for species distribution models using logistic regression pre- and post-treatment (training and testing data, respectively); and on untreated control grids and treated (resource augmented) grids during post-treatment sampling sessions.

Species	Data subset	Sensitivity	Specificity	TSS[b]	Kappa[c]	Overall Accuracy	p-value
Akodon montensis	Pre-treatment[a]	0.419	0.877	0.296	0.307	0.667	
	Post-treatment[a]	0.452	0.843	0.295	0.314	0.710	0.500
	Control	0.487	0.597	0.084	0.063	0.515	
	Treated	0.400	0.604	0.004	0.004	0.519	0.229
Oligoryzomys nigripes	Pre-treatment	0.411	0.784	0.194	0.069	0.766	
	Post-treatment	0.441	0.797	0.127	0.128	0.769	**0.001**
	Control	0.102	0.761	-0.136	-0.061	0.719	
	Treated	0.541	0.525	0.068	0.041	0.528	0.054
Hylaeamys megacephalus	Pre-treatment	0.475	0.805	0.28	0.184	0.771	
	Post-treatment	0.515	0.773	0.289	0.191	0.743	**0.005**
	Control	0.517	0.591	0.108	0.059	0.581	
	Treated	0.514	0.581	0.096	0.076	0.565	**0.000**

a. Pre-treatment data were used to train the model and post-treatment data were evaluated over all grids. b. TSS = true skill statistic. c. Cohen's kappa. d. p-value is a Student's t test with Welch's correction between the predicted probability of capturing a rodent from a given species in the sample subset pairs Pre- vs. Post-treatment or Control vs. Treated.

Table S6. Species distribution model performance. Pre-treatment collection data were used to train a species distribution model for the three most abundant species. The species distribution models were logistic regression of capture data fitted to the first six components of a principal components analysis of habitat variables measured at each trapping station on six trapping grids (863 total trap stations). To evaluate the performance of the model, the predicted presence/absence for each species was compared to the observed presence/absence for the training dataset and the post-treatment (testing) dataset. Statistics (specificity, overall accuracy, and true skill statistic) were derived from the confusion matrix. Also shown are the number of trap sites at which the two principal hantavirus reservoirs (*Akodon montensis* and *Oligoryzomys nigripes*) are predicted to co-occur with each other and with another abundant sympatric rodent (*Hylaeamys megacephalus*).

	A. montensis	***H. megacephalus***	***O. nigripes***
Total traps	863	863	863
Predicted presence	568	388	316
Cooccur with *H. megacephalus*	87	-	-
Cooccur with *O. nigripes*	65	38	-
Specificity	0.869 / 0.840	0.842 / 0.866	0.836 / 0.841
TSS[a]	0.314 / 0.284	0.236 / 0.136	0.282 / 0.113
Overall Accuracy[b]	0.674 / 0.705	0.796 / 0.776	0.817 / 0.818

a. True Skill Statistic (TSS) = Specificity+Sensitivity-1; b. sum of true positives and true negatives divided by the total.
